# Outpatient Antibiotic Prescribing Patterns in Children among Primary Healthcare Institutions in China: A Nationwide Retrospective Study, 2017–2019

**DOI:** 10.3390/antibiotics13010070

**Published:** 2024-01-10

**Authors:** Haishaerjiang Wushouer, Kexin Du, Junxuan Yu, Wanmeng Zhang, Lin Hu, Weihsin Ko, Mengyuan Fu, Bo Zheng, Luwen Shi, Xiaodong Guan

**Affiliations:** 1 Department of Pharmacy Administration and Clinical Pharmacy, School of Pharmaceutical Sciences, Peking University, Beijing 100191, China; kaiser@pku.edu.cn (H.W.); 2011110135@stu.pku.edu.cn (K.D.); 2211210069@stu.pku.edu.cn (J.Y.); 2111210065@bjmu.edu.cn (W.Z.); michelle_hlin@pku.edu.cn (L.H.); 2211110175@stu.pku.edu.cn (W.K.); mengyuan_fu@pku.edu.cn (M.F.); 2 International Research Center for Medicinal Administration (IRCMA), Peking University, Beijing 100191, China; 3 Institute of Clinical Pharmacology, Peking University First Hospital, Beijing 100034, China; doctorzhengbo@163.com

**Keywords:** antibiotic use, children, broad-spectrum antibiotics, appropriateness, diagnostic classification

## Abstract

There is scarce evidence to demonstrate the pattern of antibiotic use in children in China. We aimed to describe antibiotic prescribing practices among children in primary healthcare institutions (PHIs) in China. We described outpatient antibiotic prescriptions for children in PHIs from January 2017 to December 2019 at both the national and diagnostic levels, utilizing the antibiotic prescribing rate (APR), multi-antibiotic prescribing rate (MAPR), and broad-spectrum prescribing rate (BAPR). Generalized estimating equations were adopted to analyze the factors associated with antibiotic use. Among the total 155,262.2 weighted prescriptions for children, the APR, MAPR, and BAPR were 43.5%, 9.9%, and 84.8%. At the national level, J01DC second-generation cephalosporins were the most prescribed antibiotic category (21.0%, N = 15,313.0), followed by J01DD third-generation cephalosporins (17.4%, N = 12,695.8). Watch group antibiotics accounted for 55.0% of the total antibiotic prescriptions (N = 52,056.3). At the diagnostic level, respiratory tract infections accounted for 67.4% of antibiotic prescriptions, among which prescriptions with diagnoses classified as potentially bacterial RTIs occupied the highest APR (55.0%). For each diagnostic category, the MAPR and BAPR varied. Age, region, and diagnostic categories were associated with antibiotic use. Concerns were raised regarding the appropriateness of antibiotic use, especially for broad-spectrum antibiotics.

## 1. Introduction

The emergence of antimicrobial resistance (AMR) threatens worldwide public health [1]. Studies have shown that several factors can result in the development and spread of AMR and that the inappropriate use of antibiotics is one of the important drivers [2]. Therefore, at the call of the Global Action Plan of the World Health Organization (WHO), strategies were issued on a national and regional level to reduce inappropriate antibiotic use and further conquer AMR [3]. However, compared with high- and middle-income countries with well-designed regulations, richer health resources, and less antibiotic use, the high level of antibiotic consumption in low-income countries (LICs) raised concern, especially in primary care settings [4]. A systematic review and meta-analysis found that, even under these circumstances, scarce research provided evidence on the assessment of inappropriateness and potential improvement [4].

In China, the use of antibiotics is at a high level compared with the rest of the world [5]. To promote the judicious use of antimicrobials in these age groups, antimicrobial stewardship was promoted in China. China issued the “The National Action Plan for Curbing Bacterial Drug Resistance (2016–2020)” and became one of the first countries to publish and implement action plans among middle-income countries and LMICs [6]. However, as the largest developing country in the world, the work of curbing bacterial resistance in China still faces some difficulties, including the imbalance of antibacterial drug management between regions, urban and rural areas, and institutions; the lack of motivation for pharmaceutical enterprises to develop antibacterial drugs; and the urgent need to change the habits of people’s drug use [6,7].

Children faced an elevated risk of receiving inappropriate antibiotic prescriptions due to various factors. Inappropriate antibiotic use not only fosters the development of antibiotic resistance but also influences other critical aspects such as immunity, metabolism, and behavior. Research has revealed that antibiotic exposure in children correlates with heightened risks of atopic dermatitis, allergic symptoms, obesity, and other chronic health outcomes [8]. Moreover, several factors could lead to inappropriate antibiotic use in children, like knowledge, attitudes, and practices among pediatricians related to antimicrobial agents, self-medication from parents, and false-positive skin tests [9,10,11,12,13]. Furthermore, the problem of inappropriate antibiotic use in children raises more concerns in primary care settings in China [14]. In China, due to the informatic development limit, the current antibiotic consumption surveillance network has not covered the primary care setting. Recent retrospective cross-sectional studies focusing on pediatric antibiotic use for upper respiratory infections have revealed higher prescribing rates and a greater proportion of injection formulations in underdeveloped regions [15]. Similar conclusions were drawn in studies concerning rural primary care settings in Guangdong province [16]. Moreover, children in less developed provinces face heightened risks of antibiotic misuse, both at home and during medical consultations. Currently, there is a lack of national studies on the use of antibiotics in children in China to elucidate the potential for reducing the prescription of antibiotics [17]. Therefore, this study aims to describe the use patterns of antibiotics in children among PHIs in China and explore the possible targets of AMS intervention.

## 2. Results

### 2.1. Characteristics of Antibiotic Prescriptions for Children

Table 1 provided an overview of the outpatient antibiotic prescriptions for children in primary healthcare institutions in China between 2017 and 2019. Out of the total samples size of 155,262.2 weighted samples, 43.5% (weighted number N = 67,519.1) were prescribed antibiotics. Among these, 9.9% (weighted number N = 6663.6) were prescribed multiple antibiotics, and 84.8% (weighted number N = 57,222.5) were broad-spectrum antibiotics. APR was higher in the children aged 12–17 years than in other age groups (0–1 year, 34.4%; 2–11 years, 43.7%; and 12–17 years, 44.4%). In terms of gender, APR was nearly the same in male and female children (43.6% vs. 43.4%). Antibiotics were more frequently prescribed for children in the eastern region than in other regions (eastern region, 50.5%; central region, 41.6%; and western region, 29.2%). Prescriptions from urban areas resulted in a higher APR than those from rural areas (urban, 46.2%; rural, 40.2%). Pediatric departments accounted for the majority of prescriptions (84.7%), while other departments accounted for a higher APR (52.0% vs. 41.9%).

### 2.2. Antibiotic Prescribing at the National Level

**Figure 1:**
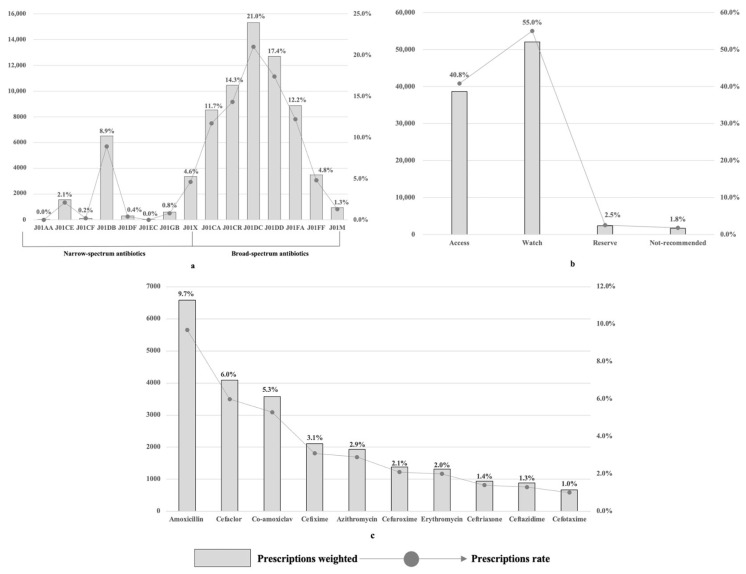
Outpatient antibiotic prescriptions for children in primary healthcare institutions at the national level. (**a**) Classified under Anatomical Therapeutic and Chemical classification; (**b**) antibiotic prescriptions under Access, Watch, Reserve classification; (**c**) top ten antibiotics prescribed for children in primary healthcare institution. Note: J01AA, tetracyclines; J01CE, beta-lactamase-sensitive penicillins; J01CF, beta-lactamase-resistant penicillins; J01DB, first-generation cephalosporins; J01DF, second-generation cephalosporins; J01EC, third-generation cephalosporins; J01GB, aminoglycosides; J01X, other antibacterials; J01CA, penicillins with extended spectrum; J01CR, combinations of penicillins; J01DC, second-generation cephalosporins, other combinations; J01DD, third-generation cephalosporins and other combinations; J01FA, macrolides, lincosamides, and streptogramins; J01FF, other macrolides and lincosamides; J01M, quinolone antibacterials.

Figure 1 demonstrated outpatient antibiotic prescriptions for children in primary healthcare institutions at the national level. J01DC second-generation cephalosporins were the most prescribed antibiotics category, accounting for 21.0% (weighted number N = 15,313.0) of total antibiotic prescriptions, followed by J01DD third-generation cephalosporins (17.4%, weighted number N = 12,695.8), J01CR combinations of penicillin, incl. beta-lactamase inhibitors (14.3%, weighted number N = 10,445.5), and J01 FA macrolides (12.2%, weighted number N = 8867.9). Broad-spectrum antibiotics accounted for 82.8% of all antibiotics prescribed. Moreover, we found the Watch group antibiotics accounted for 55.0% of total antibiotic prescriptions (weighted number N = 52,056.3) and the Access group occupied 40.8% of total antibiotic prescriptions (weighted number N = 38,624.9). Amoxicillin (9.7%), cefaclor (6.0%), and amoxicillin clavulanate potassium (5.3%) were the most prescribed antibiotics (see Tables S3 and S5).

### 2.3. Antibiotic Prescribing at Diagnostic Levels

Table 2 demonstrated outpatient antibiotic prescriptions for children in primary healthcare institutions at the diagnostic level. We found that RTIs accounted for 67.4% of antibiotic prescriptions, among which prescriptions with diagnoses classified as potentially bacterial RTIs accounted for the highest antibiotic prescribing rate (55.0%, weighted number N = 5487.1), followed by presumed viral RTIs (47.2%, weighted number N = 39,859.8) and presumed bacterial non-RTIs (45.4%, weighted number N = 12,223.5).

For each diagnostic category, the percentage of broad-spectrum antibiotic prescriptions varied. Presumed viral RTIs had the highest percentage (86.4%, weighted number N = 34,440.0), followed by presumed bacterial non-RTIs (83.6%, weighted number N = 10,218.7), and presumed bacterial RTIs (83.3%, weighted number N = 4630.3). Moreover, the practice of multi-prescribing antibiotics was observed across all diagnostic categories. For specific conditions, patients diagnosed with urinary tract infections were prescribed the most antibiotics (76.3%, weighted number N = 110.1), followed by those suffering with otitis (68.2%, weighted number N = 502.2), sinusitis (60.7%, weighted number N = 464.1), pneumonia (57.3%, weighted number N = 1880.3), and bronchitis (53.7%, weighted number N = 107.1). We found a high rate of broad-spectrum antibiotic prescription across different diagnostic categories (varied from 63.6% to 91.1%).

### 2.4. Predictors Associated with Antibiotic Prescribing

**Figure 2:**
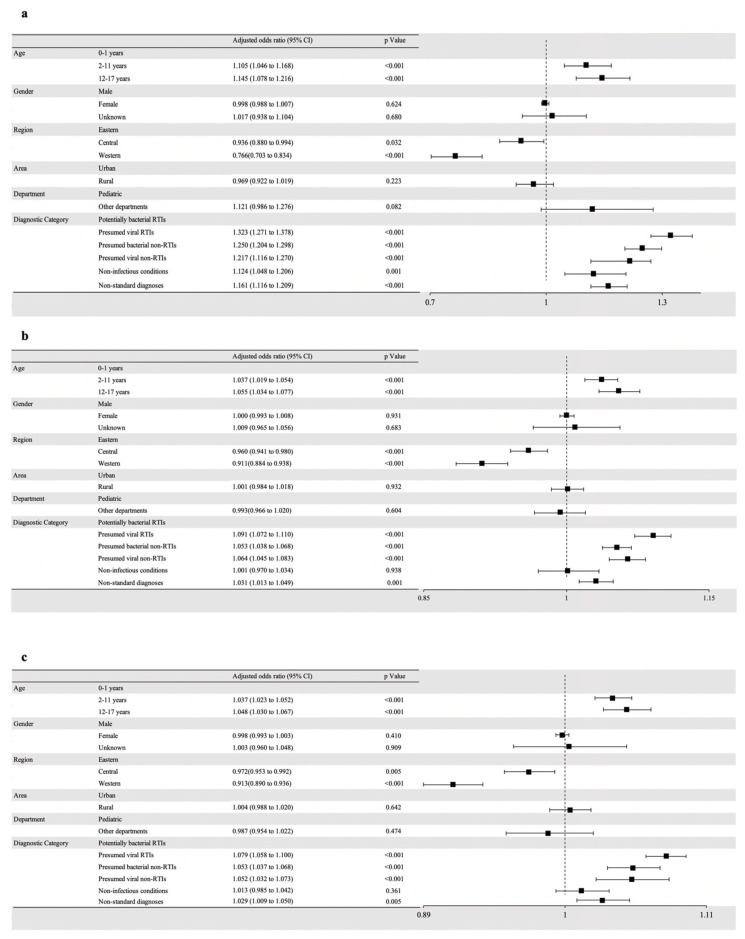
Predictors of the outpatient antibiotic prescribing for children in primary healthcare institutions in China, 2017–2019. (**a**) Predictor of antibiotic prescribing. (**b**) Predictor of multi-antibiotics prescribing. (**c**) Predictor of broad-spectrum antibiotic prescribing. Note: broad-spectrum antibiotics included combinations of penicillin with b-lactamase inhibitors (ATC J01CR), second- and third-generation cephalosporins (J01DC and J01DD), and macrolides except for erythromycin (J01F except for J01FA01).

Figure 2 showed the predictors associated with outpatient antibiotic prescriptions for children in primary healthcare institutions. Compared with neonates aged 0–1 year, older children were more likely to be prescribed with antibiotics (aOR = 1.105, *p* < 0.001 for 2–11 years, and aOR = 1.145, *p* < 0.001 for 12–17 years), multi-antibiotics (aOR = 1.037, *p* < 0.001 for 2–11 years, and aOR = 1.055, *p* < 0.001 for 12–17 years) and broad-spectrum ones (aOR = 1.037, *p* < 0.001 for 2–11 years, and aOR = 1.048, *p* < 0.001 for 12–17 years). Geographically, not only were antibiotics (aOR = 0.766, *p* < 0.001 for the western region) and multi-antibiotics more likely to be prescribed in the western region (aOR = 0.960, *p* < 0.001 for the central region, and OR = 0.911, *p* < 0.001 for the western region), but multi-antibiotics (aOR = 0.913, *p* < 0.001 for the western region) were also more likely to be prescribed in the eastern region. Compared with potentially bacterial RTIs, presumed viral RTIs, presumed bacterial non-RTIs, presumed viral non-RTIs, and non-standard diagnoses were more likely to be prescribed antibiotics (aOR = 1.323, *p* < 0.001 for presumed viral RTIs, aOR = 1.250, *p* < 0.001 for presumed bacterial non-RTIs, aOR = 1.217, *p* < 0.001 for presumed viral non-RTIs, and aOR = 1.161, *p* < 0.001 for non-standard diagnoses), multi-antibiotics (aOR = 1.091, *p* < 0.001 for presumed viral RTIs, aOR = 1.053, *p* < 0.001 for presumed bacterial non-RTIs, aOR = 1.064, *p* < 0.001 for presumed viral non-RTIs, and aOR = 1.031, *p* < 0.001 for non-standard diagnoses), and broad-spectrum antibiotics (aOR = 1.079, *p* < 0.001 for presumed viral RTIs, aOR = 1.053, *p* < 0.001 for presumed bacterial non-RTIs, aOR = 1.052, *p* < 0.001 for presumed viral non-RTIs, and aOR = 1.029, *p* < 0.001 for non-standard diagnoses). Moreover, non-infectious conditions were likely to be prescribed antibiotics compared with potentially bacterial RTIs (aOR = 1.124, *p* = 0.001).

## 3. Discussion

Our study focused on assessing primary healthcare rates of antibiotic prescription for children in China. The findings revealed an APR of 43.5% among children in these institutions. Additionally, approximately 10% of antibiotic prescriptions included more than one antibiotic, and more than 80% antibiotic prescriptions included broad-spectrum antibiotics. In comparison to other countries, our study found that the APR in PHIs for children in China was higher than those reported in France (26.1%) [18], Australia (23%) [19], the Netherlands (29%) [20], Germany (38.6%) [21], United Kingdom (36.2%) [22], Italy (8.81%) [23], and Scotland (14.2%) [24]. Several factors contribute to this relatively high prescription rate in Chinese PHIs. Studies have indicated that a low level of physician knowledge [25], inadequate adherence to guidelines [10], financial incentives [26], and time pressure [27] can contribute to higher prescription rates. In Hubei province, a study found that a substantial number of physicians in PHIs struggled with diagnosing uncertainty and consequently tended to prescribe antibiotics without sufficient clinical justification [28]. To address these issues, programs and actions have been implemented to support physicians in PHIs, including paired assistance, continued education, and training sessions to enhance their expertise [29]. However, despite the knowledge that antibiotics are not beneficial for viral upper respiratory tract infections and acute bronchitis, parental factors such as poor knowledge, perceived severity of infection, and access to non-prescription antibiotics contribute to self-medication with antibiotics (SMA) in children [30]. This phenomenon of parental SMA promotes antibiotic over-prescription in China, as children whose parents engage in SMA are more likely to be prescribed antibiotics, including intravenous antibiotics, due to parental requests [31]. Moreover, the high rate of broad-spectrum antibiotic prescribing indicated indiscriminate use of broad-spectrum antibiotics, which may be attributed to physicians’ preference for broad-spectrum antibiotics over guideline-recommended narrow-spectrum variants [12]. Factors such as low access to guidelines and reliability of experience contributed to the inappropriate choices in terms of broad-spectrum antibiotics [32]. Moreover, diagnostic tools like C-reactive protein and procalcitonin were not accessible to all PHIs due to the imbalanced medical resource distribution in China [33]. In the case of children, the physician’s choice of broad-spectrum antibiotics may be influenced by the requirement for a penicillin skin test before clinical use, as parents express concerns about intradermal injection and potential adverse reactions during the test, leading to a preference for cephalosporins [21,34].

RTIs accounted for a significant portion (67.4%) of antibiotic prescriptions in our study, with presumed viral RTIs responsible for 47.2% of all antibiotic prescriptions. This finding aligns with a previous study that demonstrated a high proportion of antibiotic prescriptions for RTIs, particularly broad-spectrum antibiotics, in PHIs [12]. Despite antibiotics not being effective for presumed viral RTIs and not recommended in guidelines, over half of prescriptions for conditions such as bronchitis and bronchiolitis, as well as more than one-third of prescriptions for a common cold, included antibiotics. These commonly encountered conditions collectively contributed to over half of all antibiotic prescriptions in primary care settings. Therefore, targeted interventions should be implemented to address presumed viral conditions, especially for bronchitis and the common cold, where antibiotics should be used sparingly. Moreover, acute pharyngitis was the most frequently diagnosed potentially bacterial RTI in our study, with an APR of 50.8%. This rate was higher than that reported in France [18] and the UK [35] and exceeded guideline recommendations, as antibiotics were only recommended for confirmed Group A streptococcal cases, which accounted for approximately 15–30% of children with sore throat [36]. This finding suggests that nearly half of the antibiotics prescribed for acute pharyngitis may be unnecessary. To improve the situation, the promotion of rapid tests for accurate bacterial infection diagnoses could be beneficial. However, implementing such measures at the national level may present challenges due to resource imbalances. Furthermore, despite guidelines (see Table S1) recommending penicillin or amoxicillin as first-line antibiotics, it should be noted that broad-spectrum antibiotics accounted for over 80% of all antibiotic prescriptions. This emphasizes the need to focus not only on the volume of antibiotics prescribed but also on the specific pattern of antibiotic use. Addressing the overuse of broad-spectrum antibiotics is crucial to ensuring effective and appropriate antibiotic treatment while minimizing the risk of AMR.

Our study revealed that children above 2 years of age had a higher risk of antibiotic prescription in primary healthcare institutions. This observation may be attributed to physicians exercising more caution with neonates due to their vulnerability to potential adverse consequences associated with antibiotic use [37]. Additionally, our findings showed that children located in the eastern regions of China were more likely to receive antibiotics. This could be a result of parents being more inclined to seek medical care for their children in the more developed eastern regions, where healthcare resources and antimicrobial stewardship measures might be more readily available compared to central and western regions, which suffer from unbalanced health resource allocation. Moreover, our study found a strong association between diagnoses and antibiotic use. Physicians tended to prescribe antibiotics for not only bacterial infections but also for other conditions that did not necessarily require antibiotics. This highlights the need for improving rational antibiotic use in primary care settings and underscores the significance of appropriate diagnosis in guiding antibiotic prescriptions. In addition, research on antibiotic prescribing has previously pointed out that diagnostic uncertainty can lead to increased reliance on antibiotics, especially for patients with respiratory symptoms [38]. Clinicians facing diagnostic uncertainty may experience anticipated regret, action bias, and risk aversion, which can result in antibiotic overprescribing [39]. Although antimicrobial stewardship interventions aim to change prescribing behavior, they may sometimes overlook clinicians’ uncertainties about diagnosis. Balancing the perceived necessity of using antibiotics to protect patients from severe infection against concerns about potential adverse consequences, including toxicity and the emergence of antimicrobial resistance, is crucial in managing diagnostic uncertainty [38]. Strategies such as watchful waiting and delayed prescribing may offer potential solutions that allow professionals to strike this balance and promote more appropriate antibiotic use in primary care settings.

Our study provides valuable insights as the first nationwide investigation of antibiotic use patterns in children within PHIs in China. Nevertheless, there are several limitations to consider in interpreting our findings. Firstly, the study sample may not fully represent all outpatient visits at PHIs across China, potentially introducing selection bias. While efforts were made to ensure the sample’s representativeness, it is essential to acknowledge that certain patient groups or regions might be underrepresented or overrepresented in our study. Secondly, the appropriateness assessment in our study focused solely on whether antibiotics were needed for specific conditions, without considering other important factors such as the route of administration, dosage, and duration of therapy. The comprehensive evaluation of appropriateness would require more detailed patient data, which are not available for this study. Thirdly, certain diagnoses may require antibiotics conditionally, based on specific clinical characteristics. Unfortunately, without access to detailed patient data, we could not fully assess the appropriateness of antibiotics for these specific cases.

## 4. Materials and Methods

### 4.1. Study Design

We conducted a nationwide retrospective observational study of prescriptions for children in PHIs in China from January 2017 to December 2019 in order to describe antibiotic prescribing among children in PHIs in China. Ethics approval was obtained from Peking University Institution Review Board (IRB00001052–17019, 24 March 2021).

### 4.2. Data Sampling and Collection

We obtained outpatient prescription data from a national survey conducted across PHIs in China. Urban community health centers and rural township hospitals were systematically selected using a two-step method from 6 out of the 31 provinces in China mainland, which were selected based on economic status. Within each selected institution, 100 outpatient prescriptions were randomly sampled from patient visits occurring on the second Tuesday of each month. Subsequently, two investigators independently extracted and cross-verified the data. Data sampling and collection were published elsewhere [10], and the data selection was detailed in Figure S1.

### 4.3. Definition

Children were defined as patients aged below 18 years old. A prescription referred to all the medicines prescribed to a patient during a single visit. Antibiotic prescriptions referred to prescriptions containing at least one antibiotic classified according to Anatomical Therapeutic and Chemical (ATC) classification J01 [40]. Narrow-spectrum antibiotics were defined as tetracyclines, narrow-spectrum penicillin, first-generation cephalosporins, aminoglycosides, sulfonamides, and nitroimidazoles. On the other hand, broad-spectrum antibiotics encompassed broad-spectrum penicillin, advanced-generation cephalosporins, macrolides, quinolones, and other antibiotics [13]. Diagnostic information was matched to the International Classification of Diseases 11th Revision (ICD–11) code through an informatics approach based on the primary diagnosis. Following the guideline for the clinical application of antibiotics [41] of China and Trinh et al.’s publication [18], all conditions in the diagnoses were classified into six categories using ICD–11 codes: (a) potentially bacterial RTIs (otitis, pharyngitis, sinusitis, and pneumonia); (b) presumed viral RTIs (bronchitis, common cold, bronchiolitis, and other viral RTIs); (c) presumed bacterial non-RTIs (skin and soft tissue infections, urinary tract infections, gastrointestinal infections, and miscellaneous bacterial infections); (d) presumed viral non-RTIs (miscellaneous viral illnesses and miscellaneous illnesses caused by other micro-organisms); (e) other conditions (Non-infectious conditions); and (f) other non-standard diagnoses (see Tables S1 and S2).

### 4.4. Measurements

We utilized various measurements to assess antibiotic use at both the national and diagnostic levels. At the national level, we employed the ATC classification and the Access, Watch, Reserve (AWaRe) classification, calculating the top 10 antibiotics to analyze the proportions of antibiotic use patterns [40,42] (see Tables S3–S5). This allowed us to gain insights into the overall patterns of antibiotic utilization. Additionally, at the diagnostic levels, we utilized the antibiotic prescribing rate (APR), multi-antibiotic prescribing rate (MAPR), and broad-spectrum prescribing rate (BAPR) to specifically describe antibiotic prescription practices. APR refers to the number of weighted prescriptions divided by the number of prescriptions with at least one antibiotic prescribed. MAPR refers to the number of antibiotic prescriptions divided by the number of prescriptions with more than one antibiotic prescribed. BAPR refers to the number of antibiotic prescriptions divided by the number of broad-spectrum prescriptions of antibiotics. These indicators provided a more detailed understanding of antibiotic use in relation to specific diagnoses.

### 4.5. Statistical Analysis

To account for any potential biases in the sample composition, we applied weighting to the prescription data, considering the distribution of economic levels and urban–rural status in the national population [43]. We further described the antibiotic prescriptions by age, gender, region (eastern, central, and western), areas of the PHIs visited (urban, rural), departments of visit, and diagnostic categories. Descriptive statistics were used to summarize the data and provide an overview of the prescription patterns. Considering the longitudinal nature of the prescription data, we employed generalized estimating equations (GEE) to identify predictors associated with APR, MAPR, and BAPR. Predictor variables were age, gender, region, areas of the PHIs visited, departments of visit, and diagnostic categories (see Table S6 for the results of multicollinearity test). A GEE model with APR, MAPR, and BAPR as the outcome variable and a logit link function was constructed. Adjusted odds ratios (aOR) with corresponding 95% confidence intervals were calculated for the predictor variables included. The statistical analysis was conducted using STATA V.15.0 (StataCorp, College Station, TX, USA) and Excel 2016.

## 5. Conclusions

Prescribing antibiotics, particularly broad-spectrum antibiotics, is a common occurrence among children in PHIs in China. The patterns and appropriateness of antibiotic use for certain clinical conditions raise concerns. Therefore, it is crucial to focus more on helping primary care settings to take steps to enhance the appropriateness of antibiotic prescriptions for children.

## Figures and Tables

**Table 1:** Characteristics of the outpatient antibiotic prescriptions for children in primary healthcare institutions in China, 2017–2019.

Characteristics	Weighted Sample Size		Prescribed ATBs		Multi-Prescribing ATBs		Broad-Spectrum ATBs	
	Prescriptions	%	Prescriptions	PR ^1^	Prescriptions	PR ^2^	Prescriptions	PR ^3^
	N = 155,262.2	100.0	N = 67,519.1	43.5%	N = 6663.6	9.9%	N = 57,222.5	84.8%
Age								
0–1 years	6559.9	4.2	2257.2	34.4%	308.1	13.6%	1973.8	87.4%
2–11 years	114,698.9	73.9	50,168.7	43.7%	4642.8	9.3%	42,942.0	85.6%
12–17 years	34,003.5	21.9	15,093.2	44.4%	1712.7	11.3%	12,306.8	81.5%
Gender								
Male	89,288.2	57.5	38,899.8	43.6%	3904.3	10.0%	33,064.6	85.0%
Female	65,789.4	42.4	28,544.3	43.4%	2757.0	9.7%	24,105.1	84.4%
Unknown	147.5	0.1	59.4	40.3%	1.12	1.9%	39.45	66.4%
Region								
Eastern	57,929.4	37.3	29,238.2	50.5%	2623.9	9.0%	23,083.7	79.0%
Central	79,351.5	51.1	33,031.9	41.6%	3416.7	10.3%	29,778.5	90.2%
Western	17,981.4	11.6	5248.9	29.2%	623.0	12.0%	4360.3	83.1%
Area								
Urban	79,202.4	51.0	36,576.4	46.2%	3659.3	10.0%	31,031.8	84.8%
Rural	76,059.8	49.0	30,942.7	40.7%	3004.2	9.7%	26,190.7	84.6%
Department								
Pediatric	131,551.1	84.7	55,177.9	41.9%	5898.4	10.7%	47,043.6	85.3%
Other departments	23,711.2	15.3	12,341.2	52.0%	765.1	6.2%	10,178.9	82.5%

Note: ATB, antibiotic; PR, prescription rate. Broad-spectrum antibiotics included combinations of penicillin with β-lactamase inhibitors (ATC J01CR), second- and third-generation cephalosporins (J01DC and J01DD), and macrolides except for erythromycin (J01F except for J01FA01). ^1^: the number of weighted prescriptions divided by the number of prescriptions with at least one antibiotic prescribed. ^2^: the number of antibiotic prescriptions divided by the number of prescriptions with more than one antibiotic prescribed. ^3^: the number of antibiotic prescriptions divided by the number of broad-spectrum prescriptions of antibiotics.

**Table 2:** Diagnostic categories of outpatient antibiotic prescriptions for children in primary healthcare institutions in China, 2017–2019.

Diagnostic Category ^1^	Conditions	Prescribed ATBs		Multi-Prescribing ATBs		Broad-Spectrum ATBs	
		Prescriptions	PR ^2^	Prescriptions	PR ^3^	Prescriptions	PR ^4^
	All conditions (n = 155,262.2)	N = 67,519.1	43.5%	N = 6663.6	9.9%	N = 57,222.5	84.8%
Potentially bacterial RTIs (n = 9977.2)		n = 5487.1	55.0%	n = 687.4	12.5%	n = 4630.3	83.3%
	Otitis (n = 736.5)	502.2	68.2%	87.6	17.4%	418.2	87.0%
	Pharyngitis (n = 5194.8)	2640.5	50.8%	202.1	7.7%	2297.2	83.0%
	Sinusitis (n = 764.4)	464.1	60.7%	39.7	8.6%	385.1	81.4%
	Pneumonia (n = 3281.5)	1880.3	57.3%	373.9	19.9%	1529.8	84.4%
Presumed viral RTIs (n = 84,514.7)		n = 39,859.8	47.2%	n = 3539.5	8.9%	n = 34,440.0	86.4%
	Bronchitis (n = 25,846.6)	13,883.5	53.7%	1357.0	9.8%	11,974.1	86.2%
	Common cold (n = 3963.9)	1331.3	33.6%	134.6	10.1%	1157.6	87.0%
	Bronchiolitis (n = 205.9)	107.1	52.0%	5.06	4.7%	97.5	91.1%
	Other viral RTIs (n = 54,498.3)	24,538.0	45.0%	2073.9	8.5%	21,210.8	86.4%
Presumed bacterial non-RTIs (n = 26,911.9)		n = 12,223.5	45.4%	n = 1336.1	10.9%	n = 10,218.7	83.6%
	Skin and soft tissue infections (n = 2418.6)	440.1	18.2%	139.89	31.8%	388.9	88.4%
	Urinary tract infections (n = 144.2)	110.1	76.3%	39.42	35.8%	99.0	90.0%
	Gastrointestinal infections (n = 6335.7)	2623.4	41.4%	232.0	8.8%	2081.9	79.4%
	Miscellaneous bacterial infections (n = 18,013.4)	9050.0	50.2%	979.5	10.8%	7649.0	84.5%
Presumed viral (Other pathogens) non-RTIs (n = 4144.9)		n = 1324.2	31.9%	n = 509.8	38.5%	n = 922.9	69.7%
	Miscellaneous viral illnesses (n = 1079.1)	384.3	35.6%	75.47	19.6%	325.3	84.6%
	Miscellaneous illnesses caused by another micro-organism (n = 3065.8)	939.9	30.7%	68.4	7.3%	597.7	63.6%
Non-infectious conditions (n = 20,557.0)		n = 4826.1	23.5%	n = 483.4	10.0%	n = 3923.4	81.3%
Non-standard diagnose (n = 9156.6)		n = 3798.4	41.5%	n = 107.4	2.8%	n = 3087.2	81.3%

Note: ATB, antibiotic; PR, prescription rate. Broad-spectrum antibiotics included combinations of penicillin with β-lactamase inhibitors (ATC J01CR), second- and third-generation cephalosporins (J01DC and J01DD) and macrolides except for erythromycin (J01F except for J01FA01). ^1^: the diagnostic categories were not mutually exclusive. ^2^: the number of weighted prescriptions divided by the number of prescriptions with at least one antibiotic prescribed. ^3^: the number of antibiotic prescriptions divided by the number of prescriptions with more than one antibiotic prescribed. The denominator (prescribed ATBs) was shown in left lines. ^4^: the number of antibiotic prescriptions divided by the number of broad-spectrum prescriptions of antibiotics. The denominator (prescribed ATBs) shown in left lines.

## Data Availability

The data that support the findings of this study will not be openly available.

## Supplementary Materials

The following supporting information can be downloaded at: https://www.mdpi.com/article/10.3390/antibiotics13010070/s1, Figure S1: The selection of antibiotic prescriptions for children in primary healthcare institutions in China; Table S1: Principles of antibiotic therapy for various bacterial infections; Table S2: Diagnostic categories classification; Table S3: Antibiotic prescribing at the national level; Table S4: AWaRe antibiotic prescribing at the national level; Table S5: Top 10 antibiotics prescribed at the national level; Table S6: Results of multicollinearity test.
