# Supplementary material for: Outpatient Antibiotic Prescribing Patterns in Children among Primary Healthcare Institutions in China: A Nationwide Retrospective Study, 2017–2019
Source: Antibiotics (Basel). 2024 Jan 10;13(1):70. doi: 10.3390/antibiotics13010070 (PMC10812453; doi:10.3390/antibiotics13010070)
Supplement: Supplementary file 1 [file antibiotics-13-00070-s001.zip › antibiotics-2752348-supplementary.pdf]

Supplementary Materials

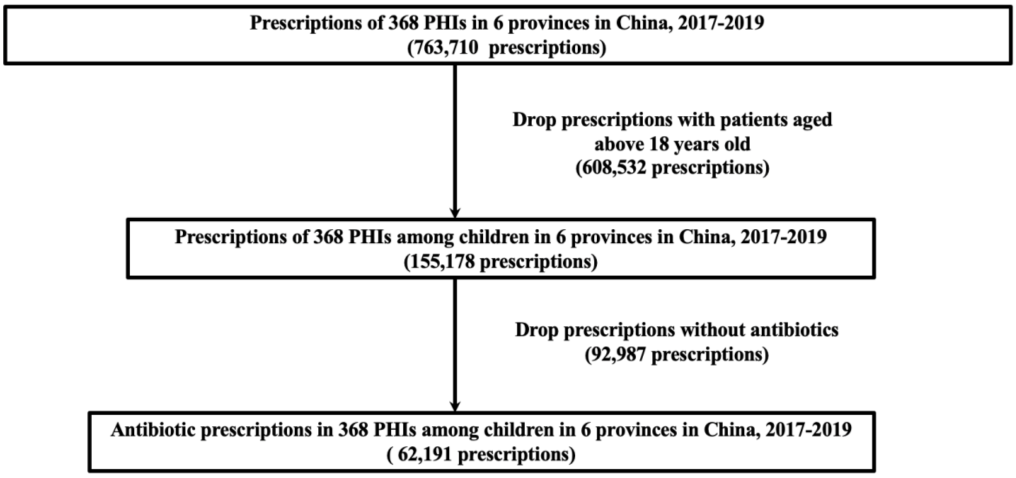

Note: PHIs, primary healthcare institutions.

Figure S1. The selection of antibiotic prescriptions for children in primary healthcare institutions in China.

Table S1. Principles of antibiotic therapy for various bacterial infections.

Condition	Recommendation
The main principle<br>Acute upper respiratory tract infection is the most common type of community-acquired infection. This is caused by a nasal virus, coronavirus, influenza, parainfluenza, or adenovirus, or sometimes caused by an intestinal virus. The course of these diseases is self-limited. They generally do not need to use of antimicrobial agents. Symptomatic illnesses can be cured, and several patients can be a primary or secondary bacterial infection based on virus infection. Antibacterial drugs are limited to the occurrence of bacterial infection symptoms, such as coughing purulent sputum or runny nose, white blood cell increase, etc. (Guidelines for clinical application of antibiotics.)	
Acute bacterial pharyngitis and tonsillitis	1. Select antibiotics for hemolytic streptococcus infection,<br>2. Penicillin is the first choice, and the course of treatment needs 10 days. Patients with penicillin allergy may take tetracycline or fluoroquinolones sensitive to hemolytic streptococcus orally. Macrolide application should refer to local drug sensitivity. Other options include oral first-or second-generation cephalosporins for 10 days but these should not be used in patients with a history of penicillin anaphylactic shock.
Acute bacterial otitis media	1. Viral upper respiratory tract infection may merge mild otitis media performance, and does not typically need antibacterial drugs. However, those characterized by acute onset of ear pain, hearing loss, fever, sexual hyperemia, and grand of the tympanic membrane, or existing tympanic membrane perforation with yellow drainage, considering the clinical diagnosis of acute bacterial otitis media, can be antibacterial treatment. The antibacterial treatment should cover streptococcus pneumonia, Haemophilus influenza, Moraxella catarrh, etc., and the course of treatment should be 7 to 10 days.<br>2. The initial treatment can be oral amoxicillin, such as for local Haemophilus influenza, and Moraxella cataract-producing β-lactamase strains are more common, and can also be treated using oral amoxicillin/clavulanate. Other options are first- or second-generation oral cephalosporins. Patients who failed to take the drug for 3 days should consider the possibility of penicillin resistance to streptococcus pneumonia infection. Oral administration of high-dose amoxicillin/clavulanate or intravenous infusion of ceftriaxone can be used. Cephalosporins should be used with caution in patients with penicillin allergy (except those with a history of penicillin anaphylactic shock). Cephalosporins should be used with caution in patients with penicillin allergy (except those with a history of penicillin anaphylactic shock).
Acute bacterial sinusitis	1. Acute bacterial sinusitis is often secondary to viral upper respiratory tract infection, most commonly involving the maxillary sinus.<br>2. Initial treatment should cover Streptococcus pneumonia, Haemophilus influenza a, and Moraxella, such as amoxicillin/clavulanate. Then, it is necessary adjust medication according to treatment response, bacterial culture, and drug sensitivity test results. The course of treatment is 10 to 14 days.

Acute trachea bronchitis	1. Antibiotics should not be routinely used. A few cases can be caused by Mycoplasma pneumonia, Bordetella pertussis, or chlamydia pneumonia, in which case antibiotics can be given. Antibiotics may be prescribed for patients with a fever over 75 years of age, patients with heart failure, insulin-dependent diabetics and patients with severe neurological disorders.<br>2. Macrolides, tetracycline, or fluoroquinolones (caused by Mycoplasma pneumonia or Bordetella pertussis). Guidelines for clinical use of doxycycline macrolides or fluoroquinolones for chlamydia p pneumonia infection
Urinary tract infection (cystitis, pyelonephritis)	1. After knowing the pathogenic bacteria and drug sensitivity test results, the adjustment should be made according to the experience of treatment effect and drug sensitivity test results.
Skin and soft tissue infections	1. Mild skin and soft tissue infections generally do not require the systemic application of antibiotics, only local use; moderate, severe, or complex skin and soft tissue infections require the systematic application of antibiotics.

Table S2. Diagnostic categories classification.

**Diagnoses**	**ICD–11 Codes**
Potentially bacterial RTIs	
Otitis	AA00;<br>AA13;<br>AA3Z;<br>AA80;<br>AA83;<br>AA8Z;<br>AA91;<br>AA9Z;<br>AA0Z;<br>AB00;<br>AB01;<br>AB0Y;<br>AB0Z;
Pharyngitis	1A61.2;<br>CA02.1Z;<br>CA02.Z;<br>CA04;<br>CA05.0;<br>CA09.2;<br>CA0G;<br>CA0K.0;<br>CA27.Z;
Sinusitis	CA00;<br>CA01;<br>CA08.Z;CA09.0;<br>CA09.1;<br>CA0A.Y;<br>CA0A.Z;
Pneumonia	CA40.0Z;CA40.1Y;<br>CA40.Z;<br>KB24;<br>L1–CA4;
Bronchitis	BD90.Z;<br>CA05.1;<br>CA20;<br>CA20.10;<br>CA20.11;<br>CA20.13;CA20.1Z;<br>CA20.Y;<br>CA20.Z;<br>CA26.0;

	CA26.Z;CA27.Z;<br>CA42.Z;<br>KB24;
Common cold	SA60;<br>L1–1E3;
Bronchiolitis	CA41.Z;<br>CA20.Y;<br>L1–EA0;
Other viral RTIs	4A80.Z<br>CA07.0;<br>CA07.1;<br>CA20.Z;CA42.Z;<br>CA45;<br>L1–CA0;
Skin and soft tissue infections	1B70.Z;<br>1B72.Z;<br>1B75.3;<br>9A06.7Z;<br>EA83;<br>EA83.0Z;<br>EA88.0;<br>EA8Z;<br>ED80.2;<br>ED90.1;<br>L1–1B7;<br>L1–EA0;<br>L2–EA8;<br>L3–EH1;
Urinary tract infections	1B12.Y;<br>GB40;<br>GB51;<br>GB55.Z;<br>GC00;<br>GC00.Z;<br>GC02.Y;<br>GC08.Z;<br>KA65.2;
Gastrointestinal infections	1A03.0;<br>1A09;<br>1A0Y;<br>1A0Z;<br>1A40.0;<br>1A40.Z;<br>9A60.Z;<br>BD90.1;<br>BD90.2Z;<br>DA42.1;<br>DA42.70<br>DA42.Y;<br>DA42.Z;<br>DA51;<br>DA97.2;<br>DB10.0;<br>DB10.Z;<br>DB33.4Z;<br>DB36.1;<br>DC50.11;L2–1A0;<br>L2–1A1;

	SA56;
Miscellaneous bacterial infections	1A70.Z;<br>1B12.6;<br>1B20.Z;<br>1C12.0;<br>1C13;<br>1C17.0Z;<br>1C20;<br>1C23.Z;<br>1D02.Y;<br>1D80;<br>1D80.0;<br>1D80.Y;<br>1E50.Z;<br>1E51.Z;<br>1F03;<br>1F05.0;<br>4A41.0;<br>4B20.1;<br>9A01.2Z;9A01.3;<br>9A02;<br>9A10.1;<br>9A11.2;<br>9A60.33;<br>9A60.4;<br>9A60.Y;<br>9A60.Z;<br>9A71;<br>9B51;<br>9C21;<br>AB0Z;<br>AB10.2;<br>AB11.0;<br>AB14;<br>BB20.Z;<br>BC42;<br>BD90;<br>BD90.0;<br>BD90.2;<br>BD90.20;<br>BD90.Y;<br>BD90.Z;<br>BD91;<br>BD93.Y;<br>BD93.Z;<br>CA02.Z;<br>CA03.0;<br>CA03.Y;CA03.Z;<br>CA0F.Z;<br>CA0K.1;DA00.0;<br>DA01;<br>DA01.0;<br>DA01.11;<br>DA01.15;<br>DA01.3;DA01.30;DA02.3;<br>DA03.0;<br>DA09.0;DA09.61;<br>DA09.6Z;

	DA09.71;
	DA09.7Z;
	DA0B;
	DA0B.4;
	DA0B.6;
	DA0B.Y;
	DA0B.Z;DA0C.0;
	DA0C.4;DA0C.5;
	DA0C.Z;
	DA24;
	DA24.0Y;
	DB10.0;
	DB10.00;
	DB10.1;
	DB10.Z;
	DB70.0Z;
	DB70.Y;
	DB70.Z;
	DC12.0;
	DC12.1;
	DC12.Z;
	DC13;
	DC1Z;
	DC31;
	EA50.2;
	EE1Z;
	EG62;
	FA10.0;
	FB40.Z;
	FB55.1;
	FB6Z;
	FB82.Y;
	GA00;
	GA00.0;
	GA00.1;
	GA00.2;
	GA01.0Z;
	GA02.0;
	GA02.2;
	GA04;
	GA05.0;
	GA05.1;
	GA05.Z;
	GA07.Z;
	GA91;
	GA91.0;GA91.Z;
	GB02.Y;
	GB02.Z;
	GB06.0Z;GB07.2;GB21.Z;KA62.9;
	KA65.1;
	L1–1B4;
	L1–1G4;L1–BD7;
	L1–EA0;
	L1–FB3;
	L2–9A7;
	MA01;
	MD31;
	ME05.1;

	ME10.1;<br>NE81.2;<br>XH5LM1;
Miscellaneous viral illnesses	1A22;<br>1A2Z;<br>1A40.Z;<br>1A94.Z;<br>1B72.Y;<br>1C8E.Z;<br>1D80.Y;<br>1D84;<br>1D85.Z;<br>1D90;<br>1D91;<br>1D9Z;<br>1E8Z;<br>1E90.0;<br>1E90.Z;<br>1E91.1;<br>1E91.3;<br>1E91.Z;<br>1F00.00;<br>1F00.01;<br>1F00.02;<br>1F00.1;<br>1F00.Z;<br>1F03;<br>1F05.0;<br>9A60.Y<br>CA02.1Y;<br>CA02.1Z;<br>CA03.Y;<br>CA07.0;<br>CA40.11;<br>CA42.1;<br>CA42.2;<br>EB44;<br>KA62.4;<br>KA62.8;<br>L1–1E5;<br>L2–1A2;<br>L3–AA0;<br>ME66.6Z;
Miscellaneous illnesses caused by another micro-organism	1A36.12;<br>1A36.Z;<br>1A3Y;<br>1A3Z;<br>1A92;<br>1C20;<br>1C2Y;<br>1E76;<br>1E81;<br>1F20.0Y;<br>1F20.Z;<br>1F23;<br>1F23.0;<br>1F23.10;<br>1F23.1Z;

	1F28;
	1F28.0;
	1F28.2;
	1F28.3;
	1F28.Y;
	1F28.Z;
	1F2C;
	1F2D.0;
	1F2D.Y;
	AB11.Z;
	CA03.0;
	CA40.04;
	CA42.Y;
	EA60.Z;
	EA83.0;
	EA90;
	EE12.1;
	KA65.Y;
	L1–1F2;
	L2–1F6;
	SB79;
	XN1W2;
Other conditions<br>Noninfectious conditions	/
Non-standard diagnosis	/

Table S3. Antibiotic prescribing at the national level.

ATC Classification	Prescriptions Weighted	Prescription Rate
Narrow-spectrum antibiotics		
J01AA	20.8	0.0%
J01CE	1556.4	2.1%
J01CF	114.0	0.2%
J01DB	6516.6	8.9%
J01DF	304.3	0.4%
J01EC	27.7	0.0%
J01GB	603.9	0.8%
J01X	3368.1	4.6%
Broad-spectrum antibiotics		
J01CA	8527.7	11.7%
J01CR	10,445.5	14.3%
J01DC	15,313.0	21.0%
J01DD	12,695.8	17.4%
J01FA	8867.9	12.2%
J01FF	3497.3	4.8%
J01M	948.0	1.3%

Table S4. AWaRe antibiotic prescribing at the national level.

AWaRe Grouping	Prescriptions Weighted	Prescription Rate
Access	38,624.9	40.8%
Watch	52,056.3	55.0%
Reserve	2354.4	2.5%
Not recommended	1687.5	1.8%

Table S5. Top 10 antibiotics prescribed at the national level.

Top 10 Antibiotics	Prescriptions Weighted	Prescription Rate
Amoxicillin	6577.3	9.7%
Cefaclor	4084.4	6.0%
Amoxicillin clavulanate potassium	3576.6	5.3%
Cefixime	2102.3	3.1%
Azithromycin	1926.6	2.9%
Cefuroxime	1388.4	2.1%
Erythromycin	1317.6	2.0%
Ceftriaxone	938.0	1.4%
Ceftazidime	880.5	1.3%
Cefotaxime	675.2	1.0%

Table S6. Results of multicollinearity test.

	VIF<br>Mean (1.05)	1/VIF
Age	1.01	0.99
Sex	1.00	1.00
Region	1.09	0.91
Area	1.08	0.93
Department	1.08	0.93
Diagnostic Category	1.01	0.99
